# Determination of the optimal mating age of colonised *Glossina brevipalpis* and *Glossina austeni* using walk-in field cages in South Africa

**DOI:** 10.1186/s13071-015-1073-1

**Published:** 2015-09-17

**Authors:** Chantel J. de Beer, Gert J. Venter, Marc J. B. Vreysen

**Affiliations:** Agricultural Research Council-Onderstepoort Veterinary Institute Parasites, Vectors & Vector-borne Diseases, Private Bag X 05, Onderstepoort, 0110 South Africa; Department of Zoology and Entomology, Faculty of Natural and Agricultural Sciences, University of the Free State, PO Box 339, Bloemfontein, 9300 South Africa; Joint FAO/IAEA Division of Nuclear Techniques in Food and Agriculture, Insect Pest Control Laboratory, PO Box 100, A-1400 Vienna, Austria

**Keywords:** Tsetse flies, Sterile insect technique, Optimal mating age, Walk-in field cage, Mating competitiveness

## Abstract

**Background:**

For the control of *Glossina brevipalpis* and *Glossina austeni* that occur in South Africa an area-wide integrated pest management (AW-IPM) program with a sterile insect technique (SIT) component has been proposed. The quality of the released sterile male tsetse flies will greatly determine the success of the SIT component of the programme. Sterile males need to be able to compete with wild males immediately after their release in the affected area. The mating competitiveness can be affected by many factors including the optimal mating age of the fly which can have an impact on the timing of the release.

**Methods:**

To assess the optimal mating age for *G. brevipalpis* and *G. austeni*, mating competitiveness studies were carried out in a walk-in field cage. First, the time of peak fly activity was determined by performing the experiment in the morning and then again in the afternoon. Thereafter, 3, 6 and 9-day-old male flies competed for 3-day-old virgin females.

**Results:**

There were no significant differences in mating performance when the field cage experiments were done in the morning or in the afternoon. However, the mating latency was shorter in the afternoon than in the morning. For both species 9-day-old males mated significantly more often than 6 or 3-day-old males. Age did not affect the males’ ability to transfer sperm, mating duration or the mating latency. All females that mated were inseminated.

**Conclusions:**

Age did influence the mating competitiveness of *G. brevipalpis* and *G. austeni* and it is recommended that sterile males are not released before the age of 9 days. Keeping the male flies in the rearing facility for 8 days will have economic and logistic consequences for AW-IPM programmes that have a SIT component.

## Background

Tsetse flies (Diptera: Glossinidae) are the vectors of the trypanosome parasites that cause African trypanosomosis, an important tropical disease affecting livestock (nagana) and humans (sleeping sickness) throughout sub-Saharan Africa [1]. Of the 31 species and subspecies present in Africa only *Glossina brevipalpis* and *Glossina austeni* occur presently in South Africa where they are responsible for the cyclical transmission of *Trypanosoma brucei brucei, Trypanosoma congolense* and *Trypanosoma vivax,* the causative agents of nagana [2–5].

The tsetse infested area (±16 000 km^2^) in South Africa is confined to the north-eastern part of KwaZulu-Natal Province. It stretches from the Mfolozi River (−28.499639, 32.40) in the south to the border of Mozambique (−26.8692, 32.8342) in the north, and from the Indian Ocean coast in the east to the iMfolozi Park (−28.33416, 31.691222) in the west [6]. The flies are mostly restricted to game reserves and rural farming areas near the reserves as these areas contain suitable vegetation and hosts [7]. The *G. brevipalpis* belt stretches from Ethiopia in northern East Africa to KwaZulu-Natal in South Africa, with infested areas in Somalia, Uganda, Kenya, Rwanda, Burundi, Tanzania, Malawi, Zambia, Zimbabwe and Mozambique [8]. *Glossina austeni* is more confined to the coastal areas of East Africa and its belt extends from Somalia into Kenya, Tanzania, Zimbabwe and Mozambique [8]. The *G. brevipalpis* and *G. austeni* populations in South Africa extending into Matutuini Province of southern Mozambique are geographically relatively isolated and represent the southernmost distribution of tsetse flies in Africa. Recently, surveys in Swaziland detected the presence of *G. austeni* in the Mlawula Game Reserve in the east of the country [9, 10].

Following the outbreak of nagana in KwaZulu-Natal in 1990, the Agricultural Research Council – Onderstepoort Veterinary Institute (ARC-OVI) was commissioned to develop a sustainable strategy that would resolve the tsetse and trypanosomosis problem in South Africa [7]. After the development of suitable trapping systems for the two species [6] and the collection of various base line data sets, a strategy was proposed that was based on area-wide integrated pest management (AW-IPM) principles [3, 11]. The proposed strategy included the suppression of the *G. brevipalpis* and *G. austeni* populations with the sequential aerosol technique [3], followed by the releases of sterile males [12] to eradicate all potential relic pockets [3].

The sterile insect technique (SIT) involves the colonisation and mass-rearing of the target species for the sterilisation of the males using ionising radiation. In order to compete with the wild males, sterile males need to be released in sufficient numbers and on a sustainable basis to achieve appropriate sterile to wild male overflooding ratios [13]. Because the sperm of the released males is sterile due to the induction of numerous dominant lethal mutations [13], the mating between sterile males and fertile virgin wild females results in no offspring [14]. When adequate proportions of the wild females mate with sterile males, there will be a lower population replacement rate, which will lead to a reduction in the density of the wild target insect population, leading eventually to potential eradication [14]. The slow reproduction rate of tsetse flies [15] makes the release of sterile males very amenable for the management of tsetse fly populations [11, 16].

The successful implementation of an AW-IPM programme with an SIT component depends on a number of prerequisites [11]; the biological quality and sexual competitiveness of the sterile males being amongst the more important ones [17]. The colony reared and released sterile males must be able to compete successfully with the wild males for mating opportunities with the wild virgin females [18]. Releasing low quality sterile males will necessitate higher release rates, require more funding and might prolong the duration of the programme potentially leading to programme failure [17]. One of the factors that can influence mating success of released sterile male tsetse is the males’ age: competitiveness of *Glossina fuscipes fuscipes, Glossina palpalis palpalis*, *Glossina palpalis gambiensis*, and *Glossina pallidipes* was significantly influenced by the age of the sterilised males [19–22].

Determination of the optimal mating age of colonised tsetse flies under natural conditions in the field will be challenging, costly, and the results might be influenced by several environmental, climatic and ecological parameters which cannot be controlled. In the past, large walk-in field cages have been successfully used as a suitable surrogate for open field studies to conduct mating compatibility, mating competitiveness and other behavioural studies for fruit flies, tsetse flies and Lepidoptera [21, 23–25]. Similar field cages have successfully been used to determine the optimal mating age for *G. f. fuscipes*, *G. p. palpalis* and *G. p. gambiensis* [19].

The SIT has never been used against *G. brevipalpis* and no data are available on the optimal mating age of this species. The eradication campaign of *G. austeni* on Unguja Island, Zanzibar used sterile male flies that were mass-reared at the Tsetse and Trypanosomiasis Research Institute (TTRI) (now named Vector & Vector-Borne Diseases Research Institute) Tanga, United Republic of Tanzania, and released on the island when 3–5 days of age. No studies were, however, carried out to assess the optimal mating age of *G. austeni*.

In preparation of a potential AW-IPM programme in South Africa that could include a SIT component, this study was carried out at the ARC-OVI to determine the optimal mating age of colonised *G. brevipalpis* and *G. austeni* using walk-in field cages.

## Methods

### Colony tsetse flies

Laboratory colonies of *G. brevipalpis* and *G. austeni* were established in 2002 at the ARC-OVI in Pretoria, South Africa using seed material from the TTRI and the Entomology Unit of the FAO/IAEA’s Laboratories in Seibersdorf, Austria (now called the FAO/IAEA Insect Pest Control Laboratory), respectively. The colony flies were maintained under standard colony conditions (23–24 °C, 75–80 % RH and subdued/indirect lighting, 12 h light/12 h dark) [26, 27]. Flies were offered a blood meal daily, consisting of abattoir collected defibrinated bovine blood using an artificial *in vitro* membrane feeding system [26, 27].

### Walk-in field cage and environmental conditions

Comparative assessment of the mating performance of *G. brevipalpis* and *G. austeni* was conducted separately in large walk-in field cages under “near-natural” conditions [21, 28]. The cylindrical field cages (Ø 2.9 m x 2.0 m) were made of polyester netting with a flat floor and ceiling and a 1.5 m potted weeping boer-bean *Schotia brachypetala* tree was placed in the middle of the cages during experiments. A zip from top to bottom sealed the entrance of the cages. The field cages were deployed in a small forest of approximately 15 x 70 m, consisting of a lane of century old chir pines (*Pinus roxburghii*) on one side and water berry trees (*Syzygium cordatum*) on the other. The forest also contained two large karee trees (*Searsia lancea*) that reduced the natural light intensity and numerous undergrowth (below 3 m) of a variety of tree species; *Hyphaene coriacea, Strelitzia nicolai, Ziziphus mucronata, Cussonia spicata, Syringa persica, Ligustrum lucidum, Melia azedarach, Dracena aletriformis* and *Jacaranda* spp. The shrub and herb foliage layer (below 0.5 m) consisted of *Cyperus rotundus, Asparagus densiflorus, Tradescantia albiflora, Alpinia* spp. as well as *Hedera helix* growing on the pine trees. The forest floor had a thick carpet of leaf litter and pine needles. Compared to the surroundings, the forest was a cool, humid area with low natural light intensity.

Throughout the experiment, temperature and relative humidity were recorded every 10 min using a DS1923-F5# Hygrochron iButton data logger. Light intensity was recorded every 15 min at the top and the bottom of the cage and at the tree level using a Major Tech MT940 light meter.

To determine the optimal mating age and time of peak mating activity 30 (3-day-old) female flies of either *G. brevipalpis* or *G. austeni* were released in the middle of the cage 5 min before releasing 90 male flies of the same species, giving a male female ratio of 3:1. An observer remained inside the cages for the entire 3-h duration of the experiment. Movements of the observer were kept to a minimum. The time of mating was recorded to determine mating latency, the mating pairs collected individually into small vials, and duration of the mating observed. Although no direct adverse effect on mating behaviour was observed when mating pairs were collected, the potential influence of this action on mating behaviour cannot be ruled out. To minimise this effect mating pairs were collected similarly in all experiments. These mating pairs were not replaced in the field cages.

The mated females were dissected the following day (flies were immobilised at −5 °C before dissection) to determine insemination rate and spermathecal value [26, 27]. The spermathecae were removed and spermathecal fill was estimated by microscopic examination, they were scored as, empty (0), quarter full (0.25), half full (0.5), three quarter full (0.75) and full (1) [29]. Female flies that did not mate were dissected to confirm their virginity. All flies remaining in the cages at the end of the experiments were collected and returned to the colony. *Glossina brevipalpis* and *G. austeni* were evaluated separately.

### Time of peak mating activity

In an initial set of experiments, the time of day at which the flies showed a peak in mating performance was determined. The performance of 9-day-old male flies with 3-day-old virgin females at a males:females ratio of 3:1 was assessed in the morning (9:00–12:00 h) and again in the afternoon (13:00–16:00 h). The experiment was replicated five times for both species in a two week period in March 2012.

### Optimal mating age

The optimal mating age of males was assessed using walk-in field cages. Three-, 6- and 9-day-old male flies (30 males of each age) competed for 30 three-day-old virgin females of the same species as the males, giving a sex ratio of 3:1 (90 males: 30 females). To discriminate between the different male age groups, the flies were marked using a dot of different colours of polymer paint on the notum [21]. The males were marked 24 h before being released in the field cage experiments. The experiments with *G. brevipalpis* were carried out in March 2012 and those with *G. austeni* in March 2013.

### Mating competitiveness indicators

The propensity of mating (PM), the relative mating index (RMI) and relative mating performance (RMP) were the mating indices used to assess the mating performance of the males in the various treatments. Propensity of mating (PM) was defined as the overall proportion of released females that mated. Relative mating index (RMI) was defined as the number of pairs of one treatment group as a proportion of the total number of matings [21]. Relative mating performance (RMP) was defined as the difference between the numbers of matings of two treatments of males as a proportion of the total number of matings [21]. In addition, the mating latency time, mating duration, insemination rate and the spermatheca fill of each mated female were determined.

### Data analysis

All data were analysed using the statistical software GraphPad Instat [30]. For the time of peak mating activity, differences in the overall proportions were analysed with Chi-square (*χ*2) analysis with the Yate’s continuity correction. The *p* value was two-sided and a relative risk, p1-p2 was also determined. Additionally an unpaired test was used to differentiate between the mating latency, mating duration and spermathecal fill means (Two- tail *p* value < 0.05 was considered as significant). Where the data passed the normality test, standard (parametric) methods were used with Welch correction. If the data was not normally distributed a nonparametric method (Mann–Whitney test) was used.

For the optimal mating age determination experiments a one-way analysis of variance (ANOVA) was used to differentiate between the relative mating index, mating latency, mating duration and spermathecal fill means (*p* value < 0.05 was considered as significant). Where the data passed the normality test, standard (parametric) methods were used and the Tukey’s test was applied. If the data was not normally distributed the nonparametric Kruskal-Wallis test was used.

## Results

### Environmental conditions

**Fig. 1:**
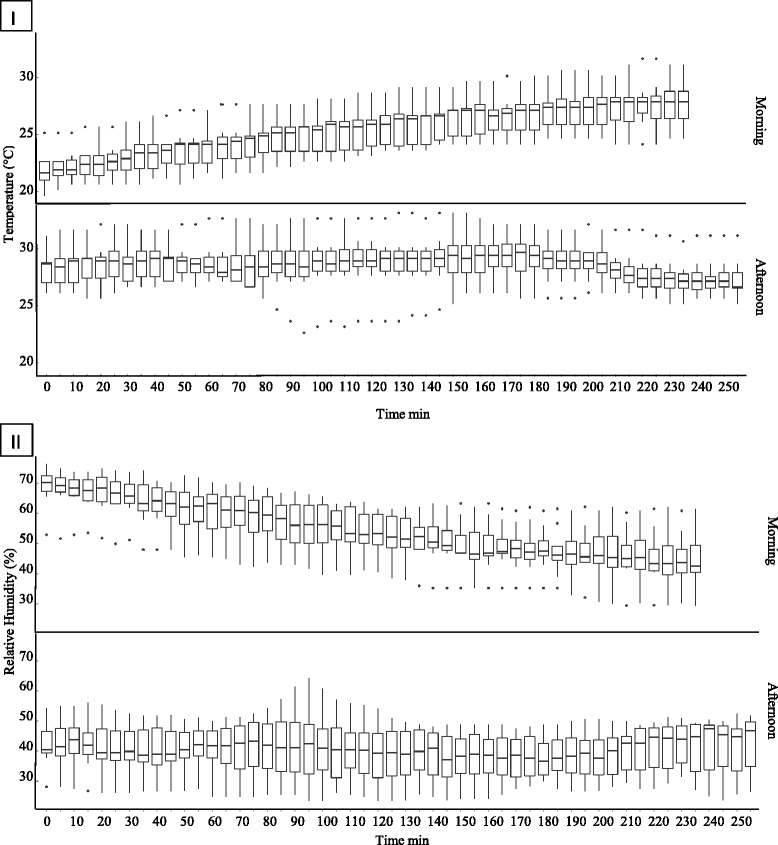
Average temperature (**i**) and average relative humidity (**ii**) recorded in field cage during the experiments in the morning and in the afternoon

All field cage experiments were conducted outdoors in a small forest at the ARC-OVI. During the ten replicates (five for each species) conducted in the morning the mean temperature gradually increased from 21.4 ± 1.4 °C at the onset of the experiments (9:00 h) to 25.0 ± 2.8 °C at the end (12:00 h) (Fig. 1i). The mean temperature in the field cages during these ten replicates was 24.4 ± 2.4 °C. The increase in temperature was accompanied by a steady decrease in relative humidity (Fig. 1ii). The RH decreased from an average of 68.0 ± 7.3 % to 52.3 ± 13.0 %, the mean being 58.6 ± 11.0 %. During the ten (five for each species) replicates conducted in the afternoon both the temperature and relative humidity were more stable. The temperature ranged from 27.6 ± 1.3 °C to 29.0 ± 2.0 °C, the mean being 28.6 ± 2.0 °C. The relative humidity ranged from 37.9 ± 7.8 % to 46.5 ± 6.8 %, the mean being 41.1 ± 4.3 %. The light intensity at the top and bottom of the cage as well as at the potted plant was in general higher in the afternoon (433.0 ± 271.6 Lx) than in the morning (301.9 ± 194.5 Lx). During the morning the light intensity was higher at the top (351.0 ± 102.7 Lx) of the cage as compared to the other areas. During the trials in the afternoon the difference in light intensity at the top (432.3 ± 155.8 Lx) and bottom (510.2 ± 387.1 Lx) of the cage was less pronounced.

### Activity in field cage

After release, males and females of both species dispersed immediately with most of the *G. brevipalpis* (males and females) settling in the top half of the cage and finding a resting site on the black band that connects the top and vertical netted panels of the cage. In contrast, male and female *G. austeni* settled mostly in the bottom half of the cage, once again favouring the black band that connects the bottom and vertical netted panels of the cage. No other differences were observed in the behaviour of *G. austeni* and *G. brevipalpis* towards the field cage environment.

For both species most of the flies settled in the more shaded areas of the cage and only a few flies settled on the tree. Some flies remained immobile after being released until being recaptured and no mating was observed for these flies. After male release, there were immediate matings, the overall minimum mating latency time was 2 min. Occasionally more than one male was trying to mate with the same female. Some attempted matings by the males were met with clear rejection from the female.

### Time of peak mating activity

**Fig. 2:**
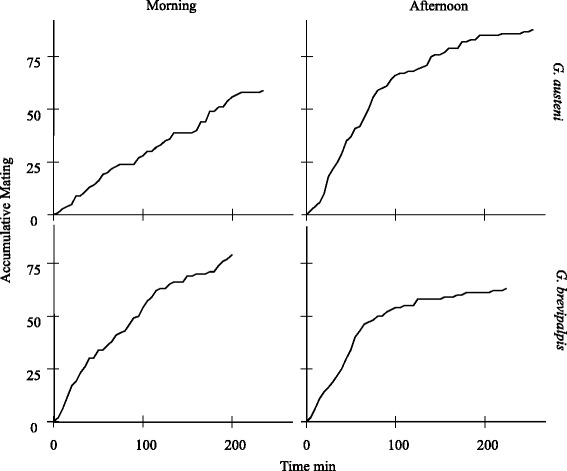
Accumulative mating for *Glossina austeni* and *G. brevipalpis* in the morning and in the afternoon

The propensity of mating in the morning was 0.70 and 0.49 for *G. brevipalpis* and *G. austeni,* respectively. This was not significantly higher than that of 0.66 for *G. brevipalpis* (*p* = 0.624) and 0.59 for *G. austeni* (*p* = 0.151) as determined in the afternoon (Table 1). The average mating latency was longer in the morning than in the afternoon for *G. brevipalpis* (*p* = 0.007) and *G. austeni* (*p* = 0.001) and was significantly different for both species (Table 1). Figure 2 indicates that for both species, more flies mated in the first hour of the experiment in the afternoon as compared with the morning experiment.

**Table 1:** Summary of various mating parameters for *Glossina brevipalpis* and *G. austeni* in the field cage for accessing the time of peak mating activity and optimal mating age

	Possible pairs	Actual mated	Overall proportion (PM)	Relative mating index (RMI ± SD)	Mating latency time (min ± SD)	Mating duration (min ± SD)	Mean spermathecal value	Insemination rate
*G. brevipalpis*								
Fly activity morning	90	63	0.70	-	73.15 ± 0.04	174.21 ± 0.06	0.75 ± 0.20	1.00
Fly activity afternoon	120	79	0.66	-	47.16 ± 0.03	165.13 ± 0.06	0.86 ± 0.12	0.99
Male age	210	97	0.46	-	54.40 ± 0.03	173.20 ± 0.03	0.72 ± 0.31	0.94
9 days	-	67	-	0.68 ± 0.23	55.03 ± 0.03	193.48 ± 0.04	0.74 ± 0.28	0.94
6 days	-	24	-	0.25 ± 0.20	56.38 ± 0.03	152.24 ± 0.04	0.79 ± 0.28	0.96
3 days	-	6	-	0.06 ± 0.06	40.33 ± 0.05	176.00 ± 0.04	0.25 ± 0.35	0.33
*G. austeni*								
Fly activity morning	120	59	0.49	-	94.33 ± 0.04	204.50 ± 0.06	0.80 ± 0.20	0.98
Fly activity afternoon	150	88	0.59	-	58.36 ± 0.04	138.63 ± 0.04	0.68 ± 0.25	0.94
Male age	360	153	0.43	-	94.30 ± 0.05	137.40 ± 0.05	0.57 ± 0.30	0.93
9 days	-	83	-	0.54 ± 0.12	97.10 ± 0.05	144.80 ± 0.06	0.61 ± 0.30	0.95
6 days	-	45	-	0.30 ± 0.16	84.07 ± 0.04	139.20 ± 0.04	0.54 ± 0.25	0.96
3 days	-	25	-	0.17 ± 0.14	103.56 ± 0.05	126.80 ± 0.04	0.51 ± 0.34	0.80

The *G. brevipalpis* couples mated on average for 174.21 ± 0.06 min in the morning and for 165.13 ± 0.06 min in the afternoon which was not significantly different (*p* = 0.558). For *G. austeni* the average mating duration in the morning (204.50 ± 0.06 min) was significant longer than in the afternoon (138.63 ± 0.04 min) (*p* < 0.001). The mean spermathecal value for *G. brevipalpis* in the morning (0.75 ± 0.20) was slightly lower than in the afternoon (0.86 ± 0.10) (*p* = 0.002), the overall insemination rate was above 99 % (Table 1). The insemination rate for *G. austeni* was above 94 %. The mean spermathecal value of *G. austeni* was significantly different (*p* = 0.003) in the morning (0.80 ± 0.20) than in the afternoon (0.68 ± 0.30) (Table 1).

### Optimal age determination

**Fig. 3:**
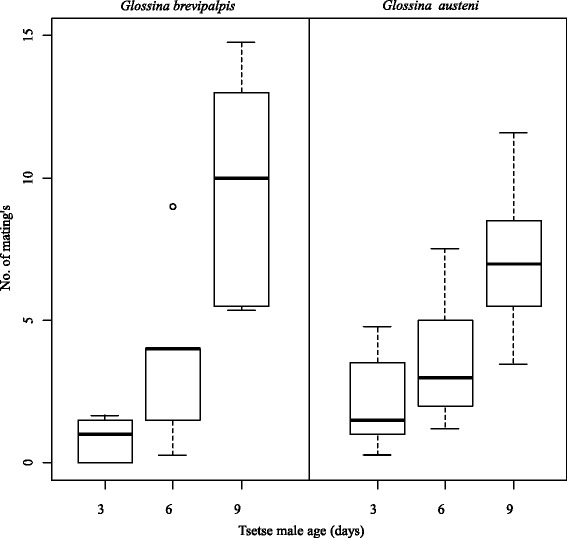
Number of males from the age groups that mated with the females in the field cage

The overall proportions of released females that mated (propensity of mating) for the optimal mating age assessment was 0.46 for *G. brevipalpis* and 0.43 for *G. austeni* (Table 1). The Relative mating performance for *G. brevipalpis* and *G. austeni* was 0.84 and 0.54 respectively and both in favour of 9-day-old males. The mean relative mating index (Table 1) for 9-day-old males (0.68 ± 0.23 for *G. brevipalpis* and 0.54 ± 0.12 for *G. austeni*) was significantly higher than that of 6-day-old (0.25 ± 0.20, *p* < 0.010 for *G. brevipalpis* and 0.30 ± 0.16, *p* < 0.010 for *G. austeni*) and 3-day-old (0.06 ± 0.06, *p* < 0.001 for *G. brevipalpis* and 0.17 ± 0.14, *p* < 0.001 for *G. austeni*) males for both species (Fig. 3). The relative mating index was not significantly different (*p* > 0.050) between 6-day and 3-day-old males for both species (Fig. 3).

For *G. brevipalpis* the mean mating latency, ranging from 40.33 ± 0.05 for 3-day-old males to 56.38 ± 0.03 for 6-day-old males was not significantly different (*p* = 0.735) (Table 1). Similarly mean mating duration ranging from 152.24 ± 0.04 for 6-day-old males to 193.48 ± 0.04 for 9-day-old males was not significantly different (*p* = 0.212) (Table 1). There were, however, significant differences in the mean spermathecal fill between age groups (*p* = 0.008). The mean spermathecal fill in 3-day-old-males (0.25 ± 0.30) was significantly different from that of 6-day-old (0.79 ± 0.30, *p* < 0.010) and 9-day-old (0.74 ± 0.30, *p* <0.050) males.

Similar for *G. austeni* the mean mating latency (ranging from 84.07 ± 0.04 for 6-day-old to 103.56 ± 0.05 for 3-day-old males) and mean mating duration (ranging from 126.80 ± 0.04 for 3-day-old to 144.80 ± 0.06 for 9-day-old males) was not significantly different (*p* = 0.444, *p* = 0.738, respectively) (Table 1). In contrast to *G. brevipalpis* no significant difference (*p* = 0.372) was observed for the different age groups (Table 1).

For *G. brevipalpis*, age did affect the insemination rate. The insemination rate for females mated with 3-day-old males was only 0.33 compare for 6-day-old (0.96) or 9-day-old males (0.94) (Table 1). For *G. austeni* the insemination rate ranged from 0.80 (3-day-old males) to 0.96 (6-day-old males) (Table 1).

## Discussion

AW-IPM programmes that include an SIT component can only be successful if the released sterile male insects are competitive with their native counterparts [17, 31]. Assessment of the mating competitiveness of the produced and released insects is therefore a prerequisite before any operational SIT programme can be initiated [11]. There are various biological (rate of development, temperature adaptation, circadian rhythm, flight capability, optimal mating age, weight, etc.) and operational (insect collection techniques, handling, radiation, release technologies, etc.) attributes that may affect the biological quality of the produced and released insect [32]. Quantification of the impact of each of these attributes on the released insects’ competitiveness is paramount to enable the development of procedures to mitigate any potential negative effects.

The results of this study indicate that the age of both *G. austeni* and *G. brevipalpis* male flies was significantly correlated with their mating performance as indicated by the RMI. Nine-day-old males were significantly more successful in securing a female for mating than 6-or 3-day-old males. These results are in agreement with data obtained for *G. f. fuscipes* and *G. p. palpalis* [19]. Although older *G. brevipalpis* and *G. austeni* males were more competitive to secure a mate in the field cages, the age of the males did not influence mating duration or insemination ability. This confirms data of Malele and Parker [33] who observed that *G. austeni* males that had mated on the day after emergence could successfully inseminate females of the same age in small laboratory cages. Our data on the optimal mating age indicate that the propensity of mating of both *G. austeni* and *G. brevipalpis* can possibly be improved by releasing older sterile males. However, this would require keeping the males longer in the rearing facility, which unavoidably will increase the maintenance and production costs. This protocol would require more blood meals to be offered to the sterile males before their release, more labour to absorb the increased handling needs and larger facilities to stockpile the flies before release. Some male thephritid fruit flies take several weeks to reach sexual maturity, and exposure to juvenile hormone mimics significantly accelerated the rate of sexual maturity in some of them [34]. In addition, adding certain supplements to the diet (e.g. protein) of the melon fly *Bactrocera cucurbitae* [35] or exposure of species such as *Bactrocera carambolae* to methyl eugenol significantly increased the mating performance of the males [36]. It would therefore be very useful to assess whether there are factors that can be implemented to shorten the period before the optimal mating age for *G. austeni* and *G. brevipalpis* is reached.

In the majority of previous control programmes that included a SIT component, the sterile males used for release were rather young, i.e. sterile male *G. austeni* were 4–7 days old when released on Unguja Island, Zanzibar [37], sterile male *G. p. palpalis* were 3–5 days old when released in the Lafia area of Nigeria [38], and sterile male *Glossina tachinoides* were 2–10 days old when released in a pilot trial in Chad [39]. Using younger males avoided losses in the rearing facility due to mortality, and was cost effective in terms of space and labour. These release protocols were in addition driven by mating observations in small laboratory cages that indicated that male mating and insemination was possible when the male flies were less than 5 days old [33], although other researchers used males that were between 5–8 days old for various experiments [22, 40, 41]. In these operational programmes, sterile males were offered at least two blood meals that contained a trypanocidal drug (e.g. 12.5 mg of Samorin.L^−1^ blood in the programme on Unguja [37]) before release that significantly reduced the risk of transmitting the disease trypanosomes.

An entirely different release strategy was used in the SIT trial against *Glossina morsitans morsitans* in the Tanga area, Tanzania, in the 1970’s. Here sterile males were released as pupae from fixed release stations and emerging males were consequently teneral and had to look for a blood meal quickly to build up energy reserves [42]. A drawback of this method was that the males were exposed to potential predation before reaching sexual maturity and could become potential vectors of the disease. Despite this, the programme was quite successful and releasing the male pupae at a rate of 135 km^−2^ resulted in a sterile male wild male overflooding ratio of 1.2:1 which, despite being low, maintained the indigenous wild fly population at the 80–95 % reduction level obtained after the initial insecticide application [43].

In our experiments, there was no significant difference in male activity and mating performance in the morning and the afternoon for both species, indicating that field cage experiments could be conducted in either the morning or afternoon at the ARC-OVI. The environmental conditions were more variable in the morning with a lower average temperature and a higher average relative humidity in contrast to the afternoon when conditions were more stable (but temperatures were on average higher and relative humidity lower). The afternoon time 12:00 to 15:00 was selected for all other field cage experiments to cover the afternoon activity peak of *G. brevipalpis*.

The equal mating performance of both *G. brevipalpis* and *G. austeni* in the afternoon and the morning seems to be conforming to the diurnal activity patterns as observed for *G. brevipalpis* in South Africa but not for *G. austeni*. These studies indicated a bimodal activity pattern for *G. brevipalpis* i.e. flies were active early in the morning from dawn until a period after sunrise and then late in the afternoon, whereas *G. austeni* showed a more pronounced unimodal activity pattern and flies were active from early morning till late afternoon [44]. The *G. austeni* data from South Africa were in contrast with those obtained by Owaga, Okelo & Chaudhury [45], who observed two *G. austeni* activity peaks in Kenya, one between 9.00 h to 10.00 h and a second between 14.00 h and 17.00 h. A study on Unguja Island showed temporal variation in the activity pattern of *G. austeni*, i.e. two activity peaks were observed in the rainy season (June/July), with the larger one around noon, and a second, smaller activity peak around 16.00 h. During the dryer and warmer period of the year (October-November), there was low activity during the day, with one peak between 15.00 h and 18.00 h. (F. Mramba, personal communication). A study carried out in September 1995 showed that sterile male *G. austeni* had an activity pattern that was very similar to that of wild insects, i.e. low activity during the day, with a pronounced peak in the afternoon (between 14.00 h and 18.00 h.) (MJB Vreysen, unpublished data).

Like most tsetse species, *G. austeni* and *G. brevipalpis* are markedly diurnal and show pronounced periodicity in their activity. Tsetse activity patterns are known to be under the control of an endogenous clock but in nature, these rhythms are influenced by environmental stimuli such as temperature and light [46]. Circadian rhythm of tsetse flies is a parameter that could have an influence on the sterile male activity in the field, and hence their competitiveness. Whereas the differences in activity patterns of *G. austeni* observed in the field may be related to different environmental conditions and stimuli, differences observed on the circadian rhythm in the laboratory are more difficult to explain. Crump & Brady [47] reported only one afternoon peak of spontaneous activity of *G. austeni* in the absence of any odours or other stimuli. Owaga, Okelo & Chaudhury [45] however, observed that the U-shaped activity pattern observed in the field persisted in the laboratory when the flies were maintained under a 12 h light; 12 h dark cycle and stable temperature and humidity conditions. The authors concluded that the activity pattern of *G. austeni* was mainly driven by endogenous factors [45].

The use of field cages to assess mating performance and mating competitiveness of important insect pests in the context of the SIT has gained considerably in importance in the last decade. Whereas originally mainly used for several species of fruit flies, its use has been expanded to other insect groups such as tsetse flies [48] and Lepidoptera [25]. Walk-in field cages have proved to be good surrogates for field studies, which are more complex to carry out and more costly. The data obtained from field cage studies are good indicators of the behaviour of reared insects, but these still need to be verified in the wild, where the released insects are competing with wild insects and are exposed to many varying stimuli. It needs to be pointed out that the field cage experiments were conducted in Pretoria which has a different climate as the tsetse infested area in KwaZulu-Natal. The different environmental conditions might have influenced the circadian rhythm, the activity patterns of the flies and the propensity of mating. It was shown in this study that field cages can be used to assess the mating performance of *G. brevipalpis* and *G. austeni* with an average propensity of mating above 46 %. Although the propensity of mating was lower than what was obtained in similar field cages with *G. f. fuscipes* and *G. p. palpalis* [19], the obtained value indicated adequate environmental conditions for the tests. This relative high propensity of mating obtained indicated that the potential interference on the mating behaviour of the flies because of the personnel intervention probably was minimal.

## Conclusions

This study indicated that the age of colonised male *G. brevipalpis* and *G. austeni* can influence their mating competiveness. For the implementation of SIT it can be recommend that sterile males of 9 days or older be released. The economic and logistic consequences of this on potential AW-IPM programmes should be taken into consideration.
